# Exploring Early and Late *Toxoplasma gondii* Strain RH Infection by Two-Dimensional Immunoblots of Chicken Immunoglobulin G and M Profiles

**DOI:** 10.1371/journal.pone.0121647

**Published:** 2015-03-24

**Authors:** Saeed El-Ashram, Ximeng Sun, Qing Yin, Xianyong Liu, Xun Suo

**Affiliations:** 1 State Key Laboratory for Agrobiotechnology, China Agricultural University, Beijing, China; 2 National Animal Protozoa Laboratory & College of Veterinary Medicine, China Agricultural University, Beijing 100193, China; 3 Key Laboratory of Animal Epidemiology and Zoonosis of Ministry of Agriculture, Beijing, China; 4 Faculty of Science, Kafr El-Sheikh University, Kafr El-Sheikh, Egypt; Henan Agricultural Univerisity, CHINA

## Abstract

*Toxoplasma gondii* is an intracellular apicomplexan parasite infecting warm-blooded vertebrate hosts, with only early infection stage being contained with drugs. But diagnosis differencing early and late infection was not available. In the present investigation, 2-dimensional immunobloting was used to explore early and late infections in chickens. The protein expression of *T*. *gondii* was determined by image analysis of the tachyzoites proteome separated by standard-one and conventional two-dimentional gel polyacrylamide electrophoresis (2D- PAGE). Pooled gels were prepared from tachyzoites of *T*. *gondii*. A representative gel spanning a pH range of 3-10 of the tachyzoite proteome consisted of 1306 distinct polypeptide spots. Two-dimensional electrophoresis (2-DE) combined with 2-DE immunoblotting was used to resolve and compare immunoglobulins (Igs) M & G patterns against *Toxoplasma gondii* strain RH (mouse virulent strain). Total tachyzoite proteins of *T*. *gondii* were separated by two-dimensional gel electrophoresis and analyzed by Western blotting for their reactivity with the 7 and 56 days post-infection (dpi) SPF chicken antisera. Different antigenic determinant patterns were detected during analysis with M and G immunoglobulins. Of the total number of polypeptide spots analyzed (1306 differentially expressed protein spots), 6.97% were identified as having shared antigenic polypeptide spots on immunoblot profiles with IgG and IgM antibodies regardless the time after infection. Furthermore, some of the immunoreactive polypeptide spots seemed to be related to the stage of infection. Interestingly, we found natural antibodies to toxoplasmic antigens, in addition to the highly conserved antigenic determinants that reacted with non-specific secondary antibody; goat anti-chicken IgG antibodies conjugated with horseradish peroxidase. In conclusion, unique reactive polypeptide spots are promising candidates for designation of molecular markers to discriminate early and late chicken infection.

## Introduction

*Toxoplasma gondii* is an intracellular apicomplexan parasite that infects a wide range of warm-blooded vertebrate hosts. The definitive hosts are members of the Felidae family including domestic and wild (feral) cats. A wide variety of vertebrates can serve as intermediate hosts including chickens. *T*. *gondii-* infected chickens are good indicators for environmental contamination with oocysts from cat feces because of their feeding habits [1– 3]. *T*. *gondii* also has been known to infect many different birds, including chickens, ducks, turkey, and ostriches [4 –11]. It has been proposed that infected chickens might function as *T*. *gondii* reservoirs and an important source of *Toxoplasma* infection [3, 6, 7]. Based chiefly on the detection of specific anti-*Toxoplasma* antibodies; several techniques were used for serodiagnosis including immunofluorescence antibody test (IFAT), immunosorbent agglutination assay (ISAGA), enzyme-linked immunofiltration assay (ELIFA), modified agglutination test (MAT), latex agglutination test (LAT), direct aglutination test (DAT), indirect hemagglutination (IHA), enzyme linked immunosorbent assay (ELISA), sabin feldman dye test (SFDT), enzyme-linked immunosorbent assay (ELISA), and immunoblotting (IB) [12, 13]. Immunoblotting techniques and enzyme immunoassays have evolved tremendously for the detection of the presence of antibodies against most of the infectious agent-specific antigens in animal and human sera [14]. The combination of toxoplasmic antigens electrophoresis under denaturing conditions, an electrotransfer, and a specific antibody examination has been exploited to compare the immunological patterns of mothers, fetuses, and infants [13, 15, 16]. The combination of the 2-DE with immunoblotting (IB) technique, namely immunoproteomics revealed many distinct antigens compared with conventional SDS-PAGE (1-DE) and its immunoblotting assay. Insight into host immunological responses against pathogen proteins has been reported by a large number of investigators using two-dimensional gel electrophoresis (2-DE) combined with antigenic proteomes [17]. The proteome and antigenic proteome (immunoproteome) have been used to explore relationships between two isolates of *Neospora caninum* (KBA-2 and VMDL-1) [18], identification of strain-specific antigens of *Toxoplasma gondii* [19], characterization of the expressed proteins of *T*. *gondii* [20, 21, 22], *Fasciola hepatica* [23], *Schistosoma japonicum* [24], *Ascaris suum* [25], and even evaluation of cross-reactivity between tachyzoites of *N*. *caninum* and *T*. *gondii* [26]. Chicken antisera raised against the rapidly dividing tachyzoite stage (invasive stage) were used in an enzyme-linked immunosorbent assay (ELISA) and Western blot (immunoblot) analysis to obtain a more detailed picture of the diagnostic polypeptide spots (molecular markers) for more precise serodiagnosis of recent and late *Toxoplasma-*infected chickens.

## Materials and Methods

### Ethics Statement

Animal experiments were conducted in accordance with the guidelines of Beijing the Municipality on the Review of Welfare and Ethics of Laboratory Animals approved by the Beijing Municipality Administration Office of Laboratory Animals (BAOLA) and under the protocol (CAU-AEC-2010–0603) approved by the China Agricultural University Animal Ethics Committee. All experimental procedures were also approved by the Institutional Animal Care and Committee of China Agricultural University (The certificate of Beijing Laboratory Animal employee, ID: 15883)

### Materials

Goat anti-chicken IgG (H+L) conjugated with horseradish peroxidase (affinity purified) were purchased from Southern Biotechnology Associate Inc., AL, USA. Fish (teleostean) gelatin was from Sigma-Aldrich, USA. Immobilon-P membranes were from Millipore (Millipore, China). Immobiline DryStrip, pH 3–10 nonlinear, 18cm; immobilized pH gradient gel (IPG) buffer and 2-D Quant Kit were from Amersham pharmacia Biotech AB, Uppsala, Sweden. Superenhanced chemiluminescent substrate (ECL) was from Shanghai Yubo Biological Technology Co, Ltd. Broad range molecular weight protein standards were from Bio-Rad; USA. All other chemicals were reagent grade.

### Experimental chickens

Two-week-old specific pathogen free (SPF) Arbor Acre (AA) broiler chicks were purchased from Beijing Arbor Acres Poultry Breeding Co., Ltd. They were housed in isolators and fed with a pathogen-free diet and water. The climatic conditions, lighting program, and chicken fodder and water were manually-operated and the chicks were cared for in agreement with the approved guidelines of the Institutional Animal Care and Committee of China Agricultural University. A total of thirty; 30-day-old chicks were randomly allocated into two groups each consisting of 10 and 20 birds. The first group consisting of chickens was served as control. The second group with 20 chickens was subcutaneously infected with 5x10^6^ live tachyzoites of *T*. *gondii* strain RH. The chickens were housed in two different isolators for the entire experiment, with food and tap water provided *ad libitum*. Chickens were exposed to a 12 h/12 h light/darkness regimen at 25°c. The chickens were not abstained from food and water before CO_2_ euthanasia.

### Blood collection and serum samples

Blood samples from experimentally infected (test) and control specific pathogen free (SPF) chicks were collected at 7 dpi (days post-infection) and 56 dpi. All the blood samples obtained from the brachial vein were allowed to clot and then centrifuged at 3000 rpm for 20 minutes; sera were aspirated, dispensed into aliquots and stored at -20°C.

### Pooled sera

Equal volumes of 9 *Toxoplasma gondii*-infected and 5 control chicken sera collected at individual dates were pooled according to collection date.

Each pool was aliquoted and the aliquots were designated 7 dpi, 56 dpi and control pooled sera respectively.

### Sample preparation and electrophoresis

#### Parasite propagation and purification

Tachyzoites of *T*. *gondii* strain RH were obtained from the peritoneal cavity of 4-day infected inbred female mice as follows: The mice were euthanized by cervical dislocation and sprayed with 70% ethanol. The outer skin of the peritoneum was removed to expose the inner skin lining of the peritoneal cavity. A peritoneal lavage with 5 ml of PBS was recovered with a syringe to collect the parasites, passed through a 27-gauge needle, and the tachyzoites were purified by sieving through 3-mm-pore-size polycarbonate filters (Millipore, China). The tachyzoite-containing filterate was centrifuged at 100 ×g for 10 minutes at 4°C to eliminate peritoneal cells and debris. In order to collect the *Toxoplasma* tachyzoites, the tachyzoite-containing supernatant then was centrifuged at 200 ×g for 10 minutes at 4°C, and the pellet was washed thrice in PBS [27, 28]. The tachyzoites from the same batch of samples were harvested, purified, washed and kept aliquots in -80 °C for experimental use.

#### One-dimensional polyacrylamide gel electrophoresis

The protein in lysis buffer (Tris-base, pH 9.6) was mixed with loading buffer at a ratio 1:5 and then boiled for 5 minutes before loading. The protein was separated using 12% gels according to Amersham protocols. The running gels were stained using 0.1% Coomassie brilliant blue R-250 in 50% methanol and 10% glacial acetic acid for 30–60 min, and then destained in 10% and 7% glacial acetic acid.

The detailed procedure for silver staining gel is described below.

#### Preparation for two-dimensional gel electrophoresis (2-DE)

*T*. *gondii* strain RH Tachyzoites were obtained from the peritoneal cavity of 4-day infected inbred female mice and protein preparations was recovered according to [28] with minor modifications as follows: Protein was prepared by rapid freezing and thawing 3 times using liquid nitrogen to disrupt tachyzoites in lysis buffer containing 7 M urea, 2 M Thiourea, 4%(W/V) CHAPS, 1%(W/V) DTT, 1mM PMSF and 0.5%(V/V) immobilized pH 3–10 gradient strips (IPG) buffer, and dissolved in 40 mM Tris-base, pH 9.6. The protein concentration was determined by 2-D Quant Kit and used at concentrations of 100μg and 300μg for standard-one and conventional two-dimentional gel polyacrylamide electrophoresis to be stained with Coomassie brilliant blue R-250. For silver staining of two-dimensional gel electrophoresis, 200μg of protein was used.

#### Two-dimensional polyacrylamide gel electrophoresis

The method was conducted according to the procedure of [28, 29] with several modifications. Tachyzoites of *Toxoplasma gondii* strain RH were subjected to non-linear immobilized pH gradient strips (IPG) using the PROTEAN IEF System—Bio-Rad. Non-linear IPG strips (pH 3–10 and 18cm) in length were rehydrated overnight with 50 μl of lysis buffer containing 200 μg or 300 μg protein (i.e. according to the staining protocol) mixed with 200 μl of rehydration sample buffer (6 M Urea; 2 M Thiourea; 4% CHAPS;); 65 mM DTT; 0.5% IPG and 0.04% w/v bromophenol blue in 40 mM Tris-base, pH 9.6).

Proteins were separated according to their isoelectric charge at 500 V for 1 hr, 1000 V for 1hr and 4500 V for 10 hrs at a constant temperature of 20°C; proteins were separated according to their isoelectric charge. IPG strips were stored at –20°C until use. The IPG strips were equilibrated for 1 hr in a reducing buffer (6 M Urea, 87% v/v Glycerol, 64.8 mM DTT, 2%w/v SDS, 0.04% Bromophenol blue and 1.5 M Tris-HCl, pH 8.8) and then followed by 1 hr in an alkylation buffer (6 M Urea, 87%v/v Glycerol, 135 mM iodoacetamide, 2% w/v SDS, 0.04% Bromophenol blue and 1.5 M Tris–HCl, pH 8.8). The equilibrated IPG sample strips were subjected to 12.5% vertical SDS-PAGE overnight at 30 mA. Broad range molecular weight protein standards were included for all protein samples. Gels were stained with Coomassie brilliant blue R-250 after fixation in 10% methanol and 7% acetic acid for 1 hr. The gels were destained twice for 1–2 hrs in 10% methanol and 7% acetic acid.

Silver staining was done essentially as previously described by [30]. After electrophoresis, gels were fixed for 2 hrs in an appropriate volume of fixing solution {(50% ethanol (or methanol), 12% acetic acid and 0.05% formalin)}. The gel was washed in 20% ethanol for 20 min after discarding the fixing solution and then incubated for 2 min in sensitizing solution (0.02% (w/v) sodium thiosulfate (Na_2_S_2_O_3_).

Next, the gels were washed twice for 1 min each, in deionized water, and incubated in cold silver staining solution {(0.2% (w/v) silver nitrate (AgNO_3_) containing 0.076% formalin)} for 20 min to allow the silver ions to bind to the polypeptide spots. In the final step, the gels were rinsed in deionized water for 20–60 sec and then in the developing solution {(6% (w/v) sodium carbonate (Na_2_CO_3_), 0.0004% (w/v) sodium thiosulfate (Na_2_S_2_O_3_) and 0.05% formalin)}. The reactions were terminated by adding 12% acetic acid.

#### Immunoblot analysis

The separated polypeptide spots from 2-DE gels were transferred to Immobilon-P Transfer Membrane (Millipore, China) for 90 min at 18 V on a Trans-blot semi-dry Transfer Cell^TM^ (Biorad) in semi-dry transfer buffer (48 mM Tris and 2.93 g glycine); pH 9.2 containing 20% methanol. The membranes were rinsed in methanol for 3 min and then washed twice with PBS-T buffer; pH 7.4 (8 mM sodium phosphate, 2 mM potassium phosphate, 140 mM NaCl, 2.7 mM KCl and 0.5% v/v Tween) for 30 s each. The blots were quenched overnight at 4°C with 1%v/v fish gelatin in PBS-T. The blotted membranes were incubated with anti- *Toxoplasma gondii* RH strain SPF chicken sera diluted 1:500 in 1%v/v fish gelatin for 1 hr at ambient temperature under constant agitation. After 3 washes in PBS-T for 15 min, the membranes were incubated with goat anti-chicken IgG (H+L chain specific) antibodies conjugated with horseradish peroxidase diluted 1:2000 in blocking buffer for 1 hr at ambient temperature under constant agitation. Membranes were washed three times with PBS-T buffer for 15 min each and one time with PBS for 10 min, then treated with two different substrate systems; superenhanced chemiluminescent substrate (ECL) plus according to the manufacturer’s instructions; the blots were exposed to X-Ray film for 30–60 s. Analysis of the 2D-immunoblotting images was carried out with PDQuestTM 2-D Analysis Software; (BIO-RAD).

## Results

**Fig 1:**
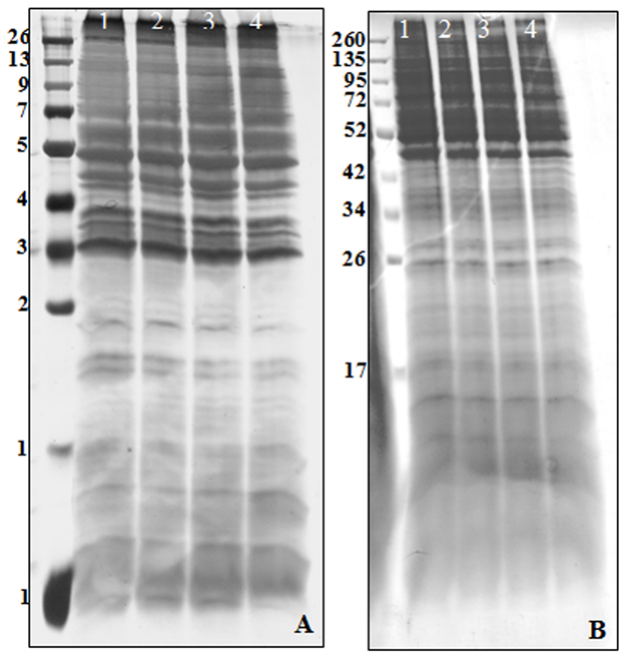
1-DE profile of *Toxoplasma gondii* strain RH tachyzoites proteins. A. Silver stained SDS-PAGE of *T*. *gondii* RH strain tachyzoite proteins separated on 12% acrylamide slab gel (the same amount of proteins were loaded and run in lanes 1–4). B. Coomassie Brilliant Blue G-250 stained SDS-PAGE of *T*. *gondii* RH strain tachyzoite proteins separated on 12% acrylamide slab gel (the same amount of proteins were loaded and run in lanes 1–4). Molecular weight standards are shown in the left hand lane.

In order to rule out environmental factors that could influence gene products, all protein preparations were conducted from the same batch of *T*. *gondii* tachyzoites for all 1D and 2D gels and blots, and the experiments were run in triplicate to avoid experimental bias and random errors. As shown in Fig. 1, the protein profile of the tachyzoite protein of *T*. *gondii* strain RH was resolved by sodium dodecyl sulfate polyacrylamide gel electrophoresis (SDS-PAGE) and stained with Coomassie brilliant blue (CBB) and silver staining methods. Figs. 1A and 1B displayed differential staining sensitivity.

**Fig 2:**
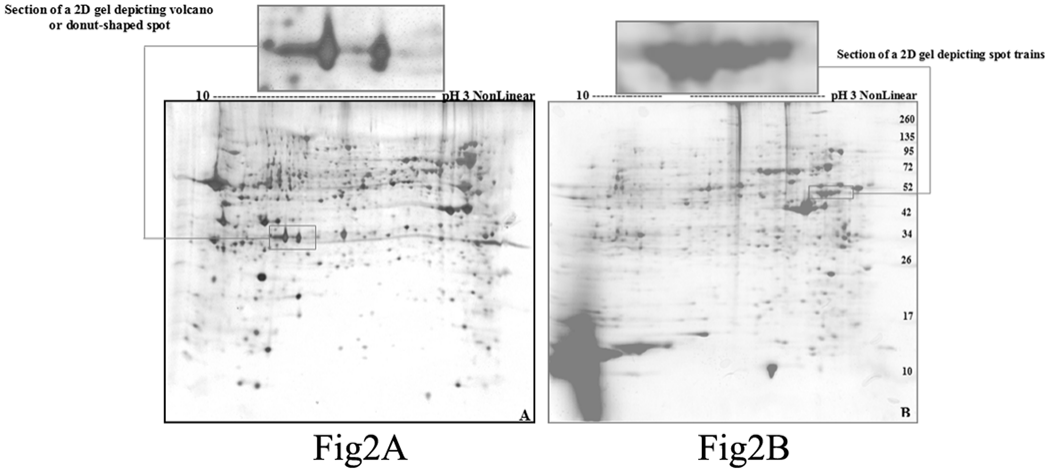
Two-dimensional reference map of the *Toxoplasma gondii* strain RH. The total tachyzoite proteins of *Toxoplasma gondii* strain RH were resolved by isoelectric focusing (IEF) and separated across the pH range 3–10 (18cm), 12% acrylamide. The polypeptide spots were visualized with silver stain. (A) and Coomassie brilliant blue G-250 (B). Molecular weight standards are shown in the right hand lane.

Although CBB stain is more convenient than the silver stain, the latter is more sensitive than CCB stain. In addition to the multi-step procedure of the silver staining method, highly expressed proteins were visualized as dark brown periphery with yellow center (i.e. volcano or donut-shaped) that can lead to problems during qualitative and quantitative analysis as in Fig. 2A. The more intense CBB and silver stain patterns of different bands that were resolved by SDS-PAGE were good indicators of the presence of more than one polypeptide in the bands at the same molecular weight or of over-expression of the same polypeptides (unpublished data) or of relative abundance of those particular polypeptides. High-resolution two-dimensional polyacrylamide gel electrophoresis (2-D PAGE) combining with the isoelectric focusing and SDS-polyacrylamide gel electrophoresis provides much better resolution than either procedure alone. The procedure separates the complex mixtures of protein extracted from biological samples according to charge (pI) by isoelectric focusing (IEF) in the first dimension and according to size (MW) by SDS-PAGE in the second dimension followed by visualizing with mass spectrometry (MS)-compatible stains (Fig. 2B). Pooled gels were prepared from tachyzoites of *T*. *gondi* strain RH. A representative gel spanning a pH range of 3–10 of the tachyzoite proteome consisted of 1306 distinct polypeptide spots.

The appearance of spot trains of differently modified and charged variants of the same polypeptide spots is pictured below (Fig. 2B). As shown in Fig. 2A, spots with yellow centers and dark boundaries could lead to problems during qualitative and quantitative analysis by image analysis software.

**Fig 3:**
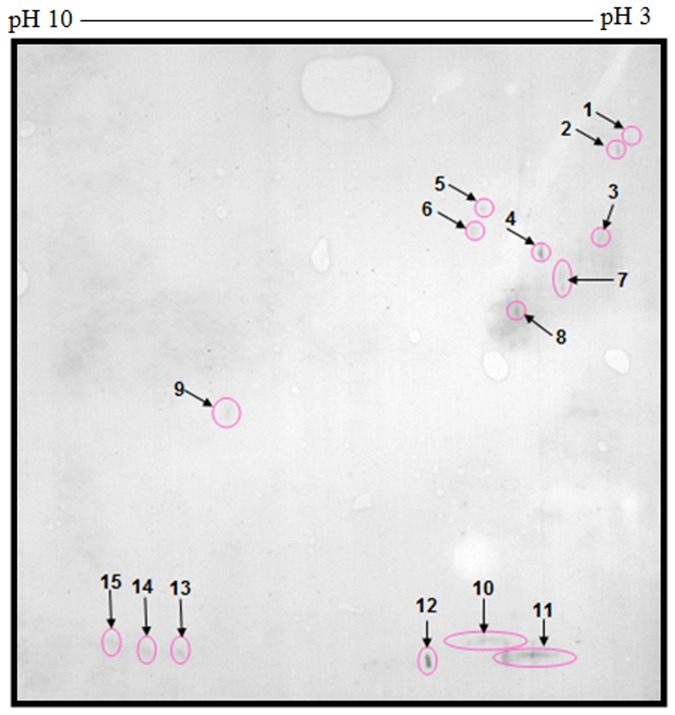
2D-immunoblotting of *T*. *gondii* strain RH tachyzoite proteins probed with pooled control sera revealed the appearance of immunoreactive polypeptide spots.

Immunological evaluations of the whole tachyzoite antigens of *T*. *gondii* strain RH were conducted. The 2-D immunoblots were probed with immune and non-immune SPF chicken sera with recent and late toxoplasmosis. Western blotting of the 2-DE gels using pooled control sera as primary antibodies and anti-chicken IgG as secondary antibodies revealed a total of 15 immunoreactive polypeptide spots (Fig. 3).

**Fig 4:**
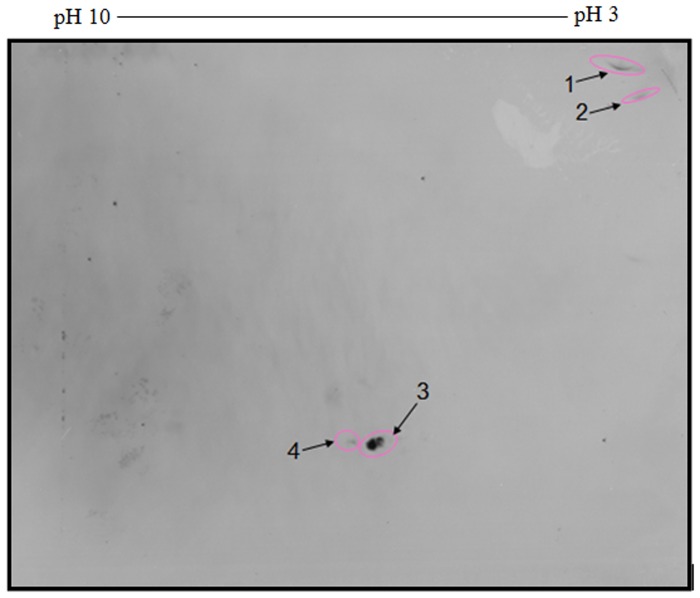
2D-immunoblotting of *T*. *gondii* strain RH tachyzoite proteins probed with goat anti-chicken IgG (H+L chain specific) antibodies conjugated with horseradish peroxidase (secondary antibody) revealed the appearance of immunoreactive polypeptide spots.

As illustrated in Fig. 4, when tachyzoite antigens of *T*. *gondii* strain RH were separated by 2-DE and electroblotted onto Polyvinylidene fluoride (PVDF) before being probed with secondary antibodies; 4 immunoreactive polypeptide spots were revealed.

The *T*. *gondii* strain RH antigenic polypeptide spots eliciting the immunoglobulin G (IgG) and IgM antibody responses were studied by using 7 dpi and 56 dpi *T*. *gondii*-infected specific pathogen free (SPF) chickens. A total of 91 shared reactive polypeptide spots were detected by using IgG and IgM antibodies, which were of lowest interest value with respect to the discrimination between early and late infection (S1 Fig.).

S1 Table provides the isoelectric point and molecular weight results of shared immunogenic polypeptide spots obtained with antibodies specific for IgG 56 dpi, IgG 7dpi, IgM 56 dpi and IgM 7dpi obtained from the PDQuestTM 2-D Analysis Software.

**Fig 5:**
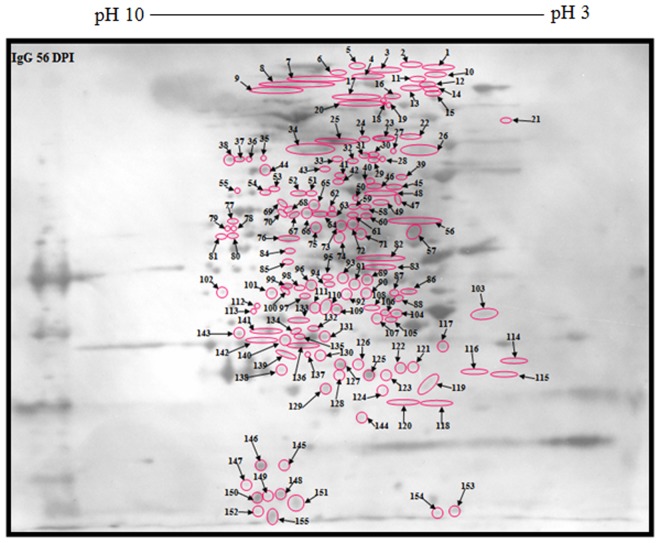
Immunoblot picture of unique reactive polypeptide spots of 2-DE separated *T*. *gondii* strain RH tachyzoite proteins using antibodies specific for IgG 56 dpi.

**Fig 6:**
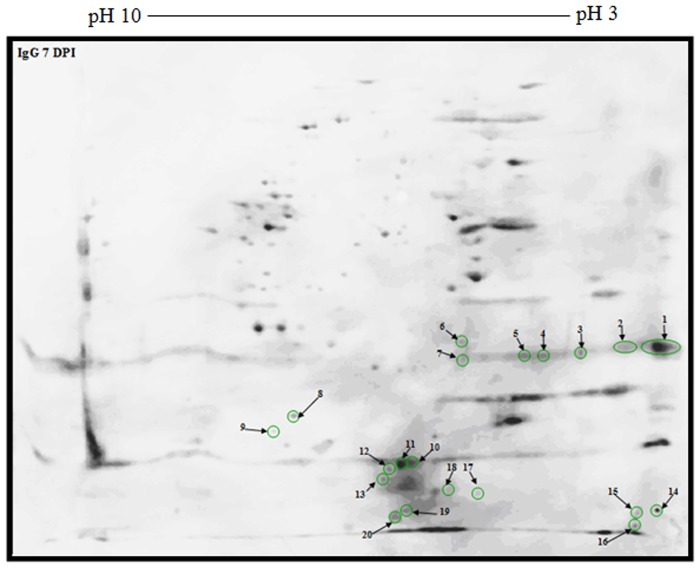
Immunoblot picture of unique reactive polypeptide spots of 2-DE separated *T*. *gondii* strain RH tachyzoite proteins using antibodies specific for IgG 7 dpi.

Moreover, IgG-*Toxoplasma* 7 dpi and 56 dpi antibodies reacted mainly with 128 polypeptide spots (S2 Fig.); however 155 polypeptide spots reacted only with IgG 56 dpi (Fig. 5). The restricted number of antigenic polypeptide spots (i.e. 20) reacting with IgG is of great diagnostic value for late infection (Fig. 6). From the data in S2 Fig, Figs. 5 and 6, the isoelectric point and molecular weight were depicted as illustrated in S2 Table, Tables 1 and 2 respectively.

**Table 1:** *T*. *gondii* strain RH tachyzoite proteins (Fig. 5).

Polypeptide spot no.	Molecular weight (Mr)	Isoelectric point (IP)	Polypeptide spot no.	Molecular weight (Mr)	Isoelectric point (IP)
**75**	60.6	6.5	**116**	12.3	4.1
**76**	59.9	6.6	**117**	15.5	4.3
**77**	61	7.2	**118**	10	4.3
**78**	60.8	7.2	**119**	11.2	4.4
**79**	60.9	7.3	**120**	10	4.5
**80**	60.2	7.2	**121**	13.3	4.4
**81**	60.1	7.3	**122**	13	4.5
**82**	37.3–39.1	4.7–5.4	**123**	12.3	4.9
**83**	30.4–30.5	5.1–5.3	**124**	11	5
**84**	46.2	6.6	**125**	12.3	5.4
**85**	34.35	6.6	**126**	13.4	5.6
**86**	24.6	4.5	**127**	13.3	6
**87**	24.5	4.6	**128**	12.3	6
**88**	23.2	4.5	**129**	11	6.4
**89**	27.2	5.5	**130**	13.7	6.4
**90**	24.3	5.5	**131**	16.7	6.4
**91**	26	5.8	**132**	17.8	6.5
**92**	23.4	5.8	**133**	19.2	6.6
**93**	27.7	6	**134**	17.4	6.6
**94**	26.3	6.3	**135**	16.6	6.6
**95**	27.7	6.3	**136**	15.6	6.6
**96**	26	6.5	**137**	15.6	6.6
**97**	23.4	6.5	**138**	12.8	6.7
**98**	25.4	6.6	**139**	14.8	6.7
**99**	25.6	6.6	**140**	16.3	6.6
**100**	24.3	6.6	**141**	17.5	6.8
**101**	24.3	6.8	**142**	16.1	6.9
**102**	24.4	7.3	**143**	17.1	7.2
**103**	20	4.1	**144**	8	5.6
**104**	20.2	4.6	**145**	5.8	6.6
**105**	19.2	4.6	**146**	5.8	6.9
**106**	20.3	5.1	**147**	5	7.1
**107**	19.4	5.3	**148**	4.6	6.7
**108**	21.3	5.4	**149**	4.6	6.8
**109**	21	6.1	**150**	4.5	6.9
**110**	21.9	6.4	**151**	4.3	6.6
**111**	20.9	6.5	**152**	4	7
**112**	21.4	7	**153**	4.4	4.2
**113**	21	7	**154**	4.1	4.4
**114**	13.6	3.9	**155**	4.2	6.8
**115**	12.1	4			

Isoelectric point and molecular weight of unique reactive polypeptide spots of 2-DE separated *T*. *gondii* strain RH tachyzoite proteins using antibodies specific for IgG 56 dpi.

**Table 2:** *T*. *gondii* strain RH tachyzoite proteins (Fig. 6).

Polypeptide spot no.	Molecular weight (Mr)	Isoelectric point (IP)	Polypeptide spot no.	Molecular weight (Mr)	Isoelectric point (IP)
**1**	10.2	3	**12**	3	5.4
**2**	10.2	3.3	**13**	2.8	5.5
**3**	9.6	3.6	**14**	2.1	3
**4**	9.3	4.1	**15**	2	3.2
**5**	9.3	4.2	**16**	1.8	3.2
**6**	10.7	4.4	**17**	2.5	4.3
**7**	9.2	4.4	**18**	2.5	4.5
**8**	5.2	6.9	**19**	2.1	5.2
**9**	4.3	7.3	**20**	1.9	5.3
**10**	3.3	5.1	**21**		
**11**	3.3	5.3	**22**		

Isoelectric point and molecular weight of unique reactive polypeptide spots of 2-DE separated *T*. *gondii* strain RH tachyzoite proteins using antibodies specific for IgG 7 dpi.

**Fig 7:**
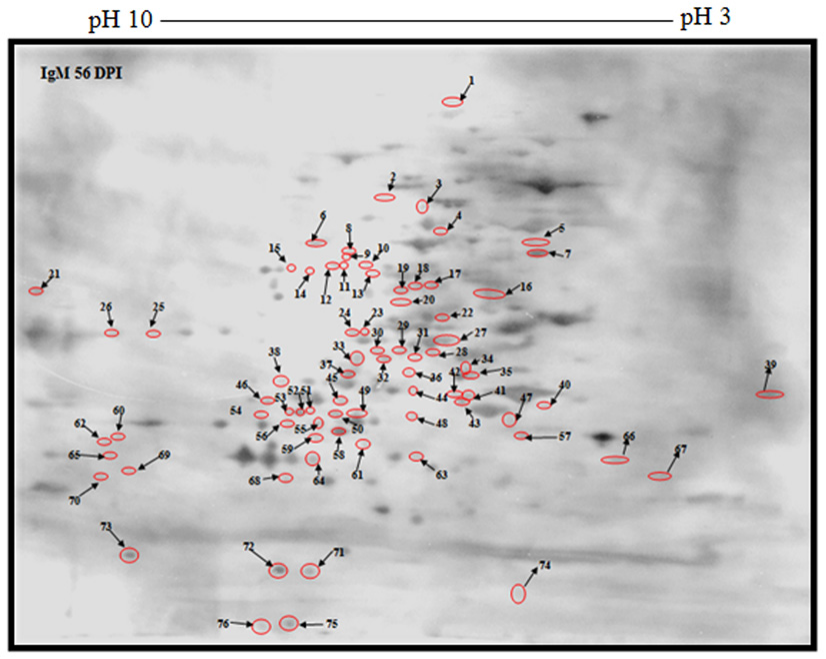
Immunoblot picture of unique reactive polypeptide spots of 2-DE separated *T*. *gondii* strain RH tachyzoite proteins using antibodies specific for IgM 56 dpi.

**Fig 8:**
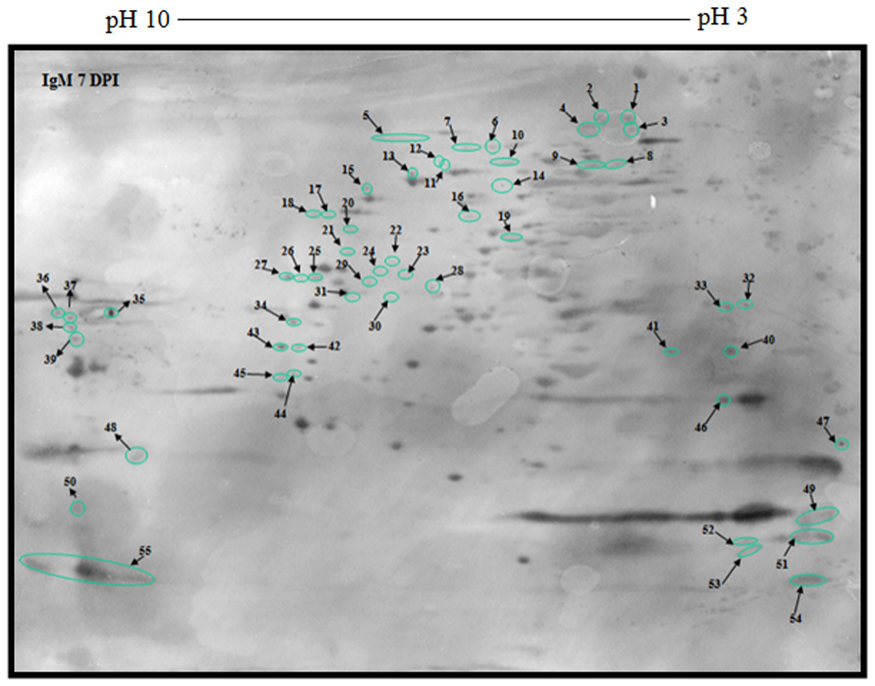
Immunoblot picture of unique reactive polypeptide spots of 2-DE separated *T*. *gondii* strain RH tachyzoite proteins using antibodies specific for IgM 7 dpi.

The serological profile of antigenic components in the IgM *Toxoplasma* antibody response was different from that of the IgG response, albeit some characteristic features were persistently observed (S3 Fig, Figs. 7 and 8). As pictured below, 145 shared IgM immunoreactive polypeptide spots were revealed by using a pool of 9 sera from 7 dpi and 56 dpi *T*. *gondii*-infected SPF chickens. However, 65 and 76 unique antigenic polypeptide spots reacted with IgM *Toxoplasma* antibodies from 7 dpi and 56 dpi SPF chickens respectively. The results of 2D-immunoblotting gels (S3 Fig, Figs. 7 and 8) that were accomplished with the aid of PDQuestTM 2-D analysis software are depicted in S3 Table, Tables 3 and 4 respectively.

**Table 3:** *T*. *gondii* strain RH tachyzoite proteins (Fig. 7).

Polypeptide spot no.	Molecular weight (Mr)	Isoelectric point (IP)	Polypeptide spot no.	Molecular weight (Mr)	Isoelectric point (IP)
**1**	329.2	5.4	**39**	21.8	3
**2**	82.7	6.5	**40**	19.3	4.3
**3**	79.4	5.8	**41**	20.2	4.6
**4**	72.8	5.4	**42**	20.3	5.1
**5**	69.6	4.3	**43**	19.2	4.6
**6**	69.6	6.8	**44**	21	5.7
**7**	66.3	4.3	**45**	19.3	6.6
**8**	66.3	6.5	**46**	18.9	7.1
**9**	66.3	6.5	**47**	17.7	4.4
**10**	63.3	6.6	**48**	17.7	5.7
**11**	63.3	6.6	**49**	17.9	6.5
**12**	63.3	6.6	**50**	17.5	6.6
**13**	61.5	6.6	**51**	19.3	6.6
**14**	62.6	6.8	**52**	19.3	6.7
**15**	62.6	7	**53**	19.3	6.8
**16**	60.2	4.4	**54**	17.2	7.2
**17**	60.8	5.7	**55**	16.3	6.6
**18**	60.8	6	**56**	17.6	6.8
**19**	60.6	6.5	**57**	15.5	4.3
**20**	60.2	6.5	**58**	16.2	6.3
**21**	59.8	9.8	**59**	14.5	6.6
**22**	47.6	5.4	**60**	16.2	8.6
**23**	30.8	7.4	**61**	13.2	6
**24**	30.8	7.5	**62**	13.2	8.7
**25**	30	8.2	**63**	13.3	5.6
**26**	30	8.6	**64**	12.8	6.7
**27**	30.4–30.5	5.1–5.3	**65**	12.8	8.7
**28**	27.2	5.5	**66**	13.6	3.9
**29**	27.8	6	**67**	10.7	3.5
**30**	27.8	6	**68**	10.7	7.1
**31**	26	5.8	**69**	10.7	8.5
**32**	26.3	6.3	**70**	10.7	8.8
**33**	26	6.5	**71**	5.7	6.7
**34**	24.5	4.6	**72**	5.7	6.9
**35**	23.2	4.5	**73**	5.9	8.5
**36**	24	5.7	**74**	4.3	4.4
**37**	23.4	6.5	**75**	3.8	6.8
**38**	21.4	7.1			

Isoelectric point and molecular weight of unique reactive polypeptide spots of 2-DE separated *T*. *gondii* strain RH tachyzoite proteins using antibodies specific for IgM 56 dpi.

**Table 4:** *T*. *gondii* strain RH tachyzoite proteins (Fig. 8).

Polypeptide spot no.	Molecular weight (Mr)	Isoelectric point (IP)	Polypeptide spot no.	Molecular weight (Mr)	Isoelectric point (IP)
**1**	343.5	4.2	**33**	52	3.6
**2**	343.5	4.2	**34**	41.8	7.4
**3**	336.2	4.3	**35**	47.4	9.4
**4**	336.2	4.3	**36**	47.3	10
**5**	96.8–98.3	6.6–6.7	**37**	44.1	10
**6**	99.8	5.2	**38**	38.8	10
**7**	99.8	5.9	**33**	52	3.6
**8**	95.7	4.2	**34**	41.8	7.4
**9**	96	4.4	**39**	32.7	9.8
**10**	95.8	4.9	**40**	28.1	3.6
**11**	96	6.2	**41**	27.9	4
**12**	95.8	6.5	**42**	29.4	7.5
**13**	89.1	6.9	**43**	29.5	7.5
**14**	91.4	4.9	**44**	20.4	7.4
**15**	89.1	6.9	**45**	18.9	7.5
**16**	79.1	6	**46**	16.3	3.6
**17**	79.1	7.1	**47**	11.3	3
**18**	79.1	7.2	**48**	10.6	9.1
**19**	70.8	5.1	**49**	6.8	3
**20**	75.5	7	**50**	4	9.6–10
**21**	68.1	7	**51**	5.4	3
**22**	63.3	6.7	**52**	5.1	3.4
**23**	61.5	6.6	**53**	4.4	3.4
**24**	62.1	6.6	**54**	3.8	3.1
**25**	61.5	7.2	**55**	3.9–4.2	9.6–10
**26**	61.5	7.3	**39**	32.7	9.8
**27**	61.5	7.5	**40**	28.1	3.6
**28**	61.1	6.5	**41**	27.9	4
**29**	61	6.9	**42**	29.4	7.5
**30**	60.3	6.7	**43**	29.5	7.5
**31**	60.3	6.9	**44**	20.4	7.4
**32**	53.7	3.5	**45**	18.9	7.5

Isoelectric point and molecular weight of unique reactive polypeptide spots of 2-DE separated *T*. *gondii* strain RH tachyzoite proteins using antibodies specific for IgM 7 dpi.

Comparative analyses of the immunologically resolved, immobilized and reactive polypeptide spots of *T*. *gondii* that have been detected by IgM and IgG *Toxoplasma* antibodies from experimentally infected SPF chickens are summarized below (Table 5).

**Table 5:** Percentage of the immunoreactive polypeptide spots.

Antibody class	%Unique 7 dpi	%Unique 56 dpi	%Shared (7 dpi &56 dpi)	%Shared all
**IgM**	4.2	5.82	11.1	6.97
**IgG**	11.9	1.53	9.8	

Further analysis of the 2-DE immunoblot profiles revealed that the signal was slightly more intense at 7 dpi than 56 dpi. This observation might be related to antibodies avidities occurring during the course of infections. From our unpublished data, the IgG antibody titers assayed by modified agglutination test (MAT) to *T*. *gondii* RH strain-infected chickens started to increase by 1 week after subcutaneous infection and peaked between the third and fourth weeks, and subsequently antibody titres declined slightly.

## Discussion

The infectious tachyzoite stage of *T*. *gondii* RH strain is the disease-causing stage in the life stage of *T*. *gondii*. Infection of SFP chickens with the *T*. *gondii* RH strain induced production of *Toxoplasma*-specific antisera which were exploited to find specific molecular markers for more precise serodiagnosis of different stages of infection (i.e. recent versus late *Toxoplasma* infection). The protein expression profile of *T*. *gondii* was determined by image analysis of the tachyzoites proteome separated by standard-one and conventional two-dimentional gel polyacrylamide electrophoresis (2D-PAGE), and the macro- and micro- scale gels of the tachyzoite proteome were stained with Coomassie brilliant blue and the high sensitive silver stain. Coomassie brilliant blue is more convenient, reproducible and an endpoint procedure compared with the multi-step silver stain procedures. Furthermore, variations in the efficacy silver staining for some proteins were reported by Morozov, et al. in 1986 [31], so this variation together with protein load variations (300μg of protein for Coomassie brilliant blue R-250 versus 200μg of protein for silver staining) could account for other differences in the visual data occurring between the two stains.

The close proximity of molecular weight bands that bear the same charge and the possibility for the presence of more than one different polypeptide in the same band in the electrophoretic profile of the *T*. *gondii* RH strain resolved by macro-scale SDS-PAGE technique led to our use of a more highly sophisticated micro-scale separating method (2D SDS-PAGE). For this purpose, pooled gels were prepared from tachyzoites of *T*. *gondii*, and a representative gel spanning a pH range of 3–10 of the tachyzoite proteome consisted of 1306 distinct polypeptide spots. More than 163 and 150 protein spots from *T*. *gondii* strains RH and KI-1 tachyzoites, in corresponding order, were detected by 2-DE within a pH range of 3–10 after silver staining [32]. A high-resolution 2-DE gel separation over pH ranges 4–7 and 6–11 was also exploited to distinguish over 1000 polypeptides spots [20], and in a separate study, 1,227 protein spots of *T*. *gondii* soluble tachyzoite antigens were resolved through fractionation by 2-DE at a pH range of 3–10 [33].

These differences could be due to sample preparation and minor differences that affect the intensities and numbers of polypeptide spots in the two-dimensional gels.

The tachyzoite immunogenic polypeptide spot analysis recognized by specific humoral immune responses has important implications for immunodiagnosis, immunotherapy and vaccination strategies. Two-dimensional electrophoresis (2-DE) combined with 2-DE immunoblotting was also used to resolve and compare M and G immunoglobulin patterns of the *Toxoplasma gondii* strain RH (mouse virulent strain). The subcutaneous route of acquisition of *T*. *gondii* strain RH appeared to be the reason the immunoglobulin A (IgA) antibody response was of low interest. Nevertheless, amongst the 1306 resolved polypeptide spots, 91 common spots were reacted with *T*. *gondii* IgM and IgG antibodies regardless of the course of infection. Furthermore, some of the immunoreactive polypeptide spots appeared to be related to the stage of infection.

During the early stage of infection (i.e. 7 days post-infection), 55 and 155 of unique polypeptide spots reacted with *T*. *gondii* IgM and IgG antibodies, respectively. However, 76 and 20 unique polypeptide spots were reacted with *T*. *gondii* IgM and IgG antibodies recovered from the late stage of infection (i.e. 56 days post-infection) in corresponding order. The unique polypeptide spots are of special interest for discrimination between early and late infections, and the early unique polypeptide spots appear to be good early infection markers. However, some of late stage spots could be good markers to discriminate late infections. Naturally occurring immunoglobulin (Ig) G antibodies to *T*. *gondii* strain RH were also observed in a pool of control sera collected from SPF chickens. Our present findings agree with those observed in previous studies [34–36], and our results are also consistent with a previous report [37] describing the presence of naturally occurring immunoglobulin M antibodies to *T*. *gondii* in Japanese populations. The existence of IgM and IgG antibodies in humans infected with *T*. *gondii* was described in the previous work [38, 39]. Further supporting evidence for the presence of natural antibodies of IgM and IgG classes against killed tachyzoites comes from fluorescence or electron microscopy studies showing localized intense staining of tachyzoites [40–42]. Moreover, goat anti-chicken IgG (H+L chain specific) antibodies conjugated with horseradish peroxidase detected 4 polypeptide spots. These spots may contain some highly conserved antigenic determinants.

In summary, an implication of our findings is that unique reactive polypeptide spots are promising candidates for the formulation of molecular markers to segregate early and late chicken infections. Furthermore, the findings of our study together with those we are currently undertaking provide information regarding a wide variety of warm-blooded vertebrate hosts. The common unique determinants can be used to develop universal chimeric molecular markers for detection of all serologically positive warm-blooded vertebrate hosts and to differentiate between different toxoplasmosis infection stages.

## Supporting Information

S1 Fig Immunoblot pictures of 2-DE separated *T*. *gondii* strain RH tachyzoite proteins using antibodies specific for the two-immunoglobulin classes; IgM and IgG. A. 2-DE immunoblot with IgM antibodies 7 dpi. B. 2-DE immunoblot with IgM antibodies 56 dpi. C. 2-DE immunoblot with IgG antibodies 7 dpi. D. 2-DE immunoblot with IgG antibodies 56 dpi.
(TIF)

S2 Fig Two-dimensional immunoblot of shared immunogenic polypeptide spots of *T*. *gondii* strain RH tachyzoite proteins using antibodies specific for IgG. A. 2-DE immunoblot with IgG antibodies 7 dpi. B. 2-DE immunoblot with IgG antibodies 56 dpi.
(TIF)

S3 Fig Two-dimensional immunoblot of shared immunogenic polypeptide spots of *T*. *gondii* strain RH tachyzoite proteins using antibodies specific for IgG. A. 2-DE immunoblot with IgM antibodies 7 dpi. B. 2-DE immunoblot with IgM antibodies 56 dpi.
(TIF)

S1 Table *T*. *gondii* strain RH tachyzoite proteins (S1 Fig.). Isoelectric point and molecular weight of shared immunogenic polypeptide spots using antibodies specific for IgG 56 dpi, IgG 7dpi, IgM 56 dpi and IgM 7dpi.
(DOC)

S2 Table *T*. *gondii* strain RH tachyzoite proteins (S2 Fig.). Isoelectric point and molecular weight of shared immunogenic polypeptide spots using antibodies specific for IgG 56 dpi and 7dpi.
(DOC)

S3 Table *T*. *gondii* strain RH tachyzoite proteins (S3 Fig.). Isoelectric point and molecular weight of shared polypeptide spots of 2-DE separated *T*. *gondii* strain RH tachyzoite proteins using antibodies specific for IgM 56 dpi and IgG 7 dpi.
(DOC)
