# Supplementary figures and images for: Exploring Early and Late Toxoplasma gondii Strain RH Infection by Two-Dimensional Immunoblots of Chicken Immunoglobulin G and M Profiles
Source: PLoS One. 2015 Mar 24;10(3):e0121647. doi: 10.1371/journal.pone.0121647 (PMC4372353; doi:10.1371/journal.pone.0121647)

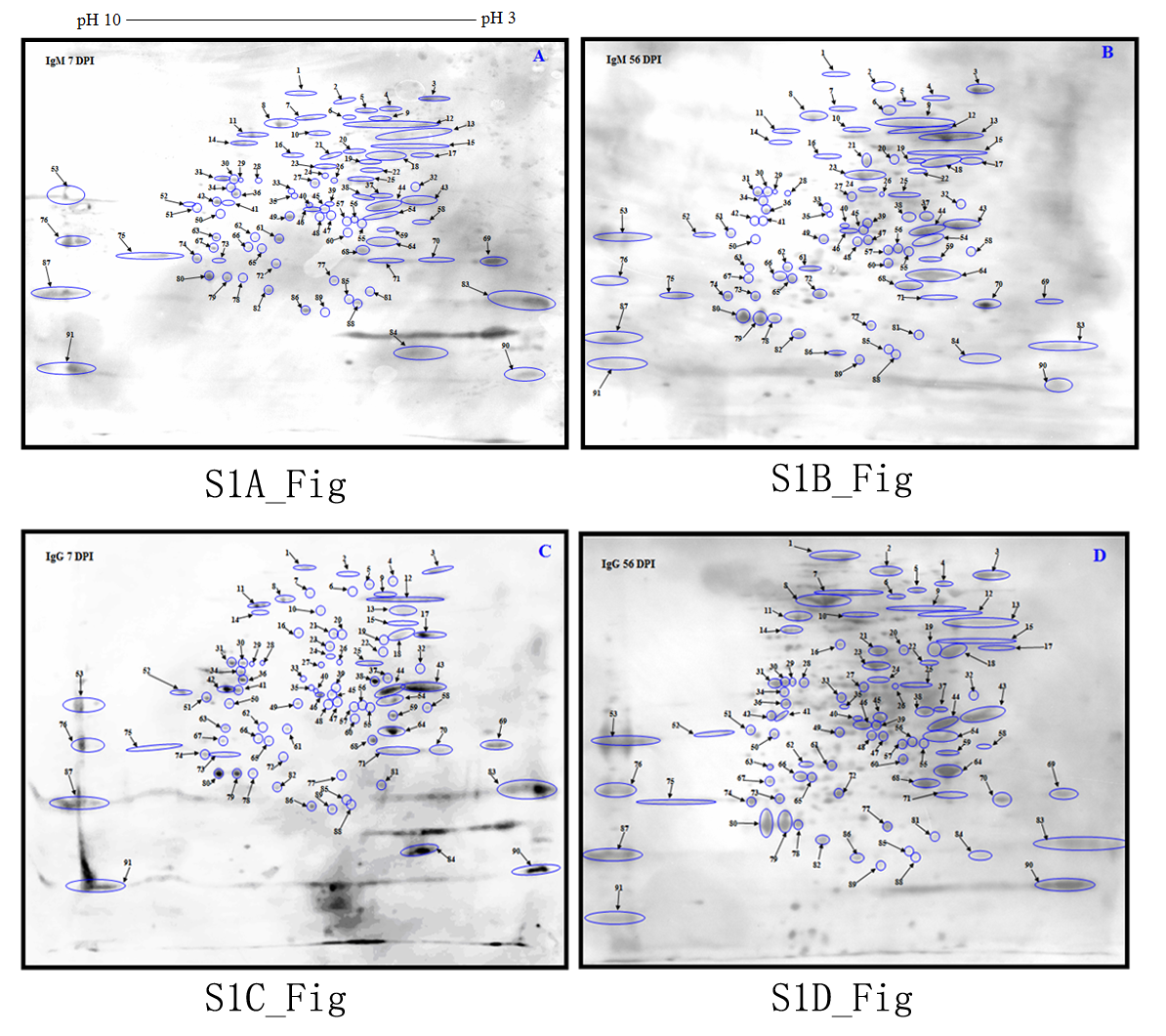

Supplement: S1 Fig — A. 2-DE immunoblot with IgM antibodies 7 dpi. B. 2-DE immunoblot with IgM antibodies 56 dpi. C. 2-DE immunoblot with IgG antibodies 7 dpi. D. 2-DE immunoblot with IgG antibodies 56 dpi. (TIF) [file pone.0121647.s001.tif]

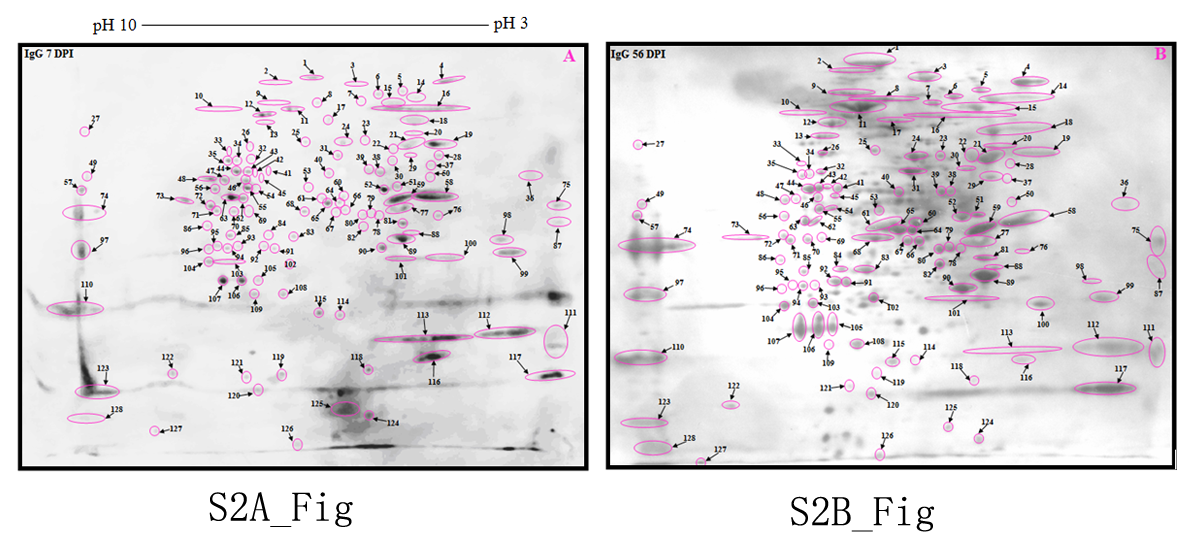

Supplement: S2 Fig — A. 2-DE immunoblot with IgG antibodies 7 dpi. B. 2-DE immunoblot with IgG antibodies 56 dpi. (TIF) [file pone.0121647.s002.tif]

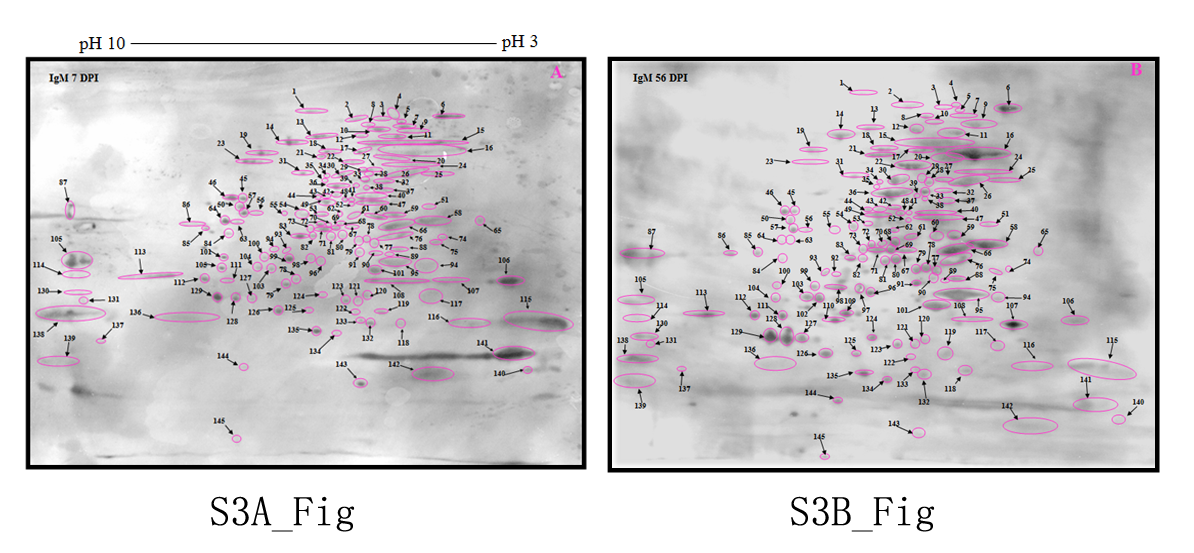

Supplement: S3 Fig — A. 2-DE immunoblot with IgM antibodies 7 dpi. B. 2-DE immunoblot with IgM antibodies 56 dpi. (TIF) [file pone.0121647.s003.tif]
