# Supplementary material for: Exploring Early and Late Toxoplasma gondii Strain RH Infection by Two-Dimensional Immunoblots of Chicken Immunoglobulin G and M Profiles
Source: PLoS One. 2015 Mar 24;10(3):e0121647. doi: 10.1371/journal.pone.0121647 (PMC4372353; doi:10.1371/journal.pone.0121647)
Supplement: S1 Table — Isoelectric point and molecular weight of shared immunogenic polypeptide spots using antibodies specific for IgG 56 dpi, IgG 7dpi, IgM 56 dpi and IgM 7dpi. (DOC) [file pone.0121647.s004.doc]

**Table S**1

| **Polypeptide spot no.** | **Molecular weight (M_r_ )** | **Isoelectric point (IP)** | **Polypeptide spot no.** | **Molecular weight (M_r_ )** | **Isoelectric point (IP)** |
| --- | --- | --- | --- | --- | --- |
| **1** | 351.5-355.8 | 6.3-6.6 | **38** | 55.8 | 4.5 |
| **2** | 309.2 | 5.4 | **39** | 47.6 | 5.7 |
| **3** | 110.8 | 4.1 | **40** | 40.5 | 6.5 |
| **4** | 106.3 | 4.3 | **41** | 50.6 | 7.1 |
| **5** | 102.7 | 4.5 | **42** | 51 | 7.2 |
| **6** | 99.2 | 5.3 | **43** | 47.2 | 4.2 |
| **7** | 100.7-101.5 | 5.8-6.4 | **44** | 37.5 | 4.4 |
| **8** | 96.8-98.3 | 6.6-6.7 | **45** | 36.3 | 5.7 |
| **9** | 96.9 | 4.4 | **46** | 37.6 | 6 |
| **10** | 90.2 | 6.2 | **47** | 29.2 | 5.5 |
| **11** | 88.7 | 6.9 | **48** | 29.4 | 5.8 |
| **12** | 92.4-92.7 | 4.5-5.7 | **49** | 30.1 | 6.5 |
| **13** | 85.1-87 | 3-4.3 | **50** | 40.3 | 7.2 |
| **14** | 82.8 | 7 | **51** | 41.8 | 7.4 |
| **15** | 78.2 | 4.3 | **52** | 45.2 | 7.5 |
| **16** | 76.8 | 6.5 | **53** | 26.7-28.3 | 9-10 |
| **17** | 75.7 | 3-4 | **54** | 28.3 | 4.4 |
| **18** | 73.4 | 4.3 | **55** | 26.4 | 4.5 |
| **19** | 69.6 | 4.5 | **56** | 27.2 | 4.5 |
| **20** | 74.1 | 4.4 | **57** | 27 | 4.8 |
| **21** | 74.8 | 4.7 | **58** | 27.2 | 4.1 |
| **22** | 65.4 | 4.5 | **59** | 24.6 | 4.3 |
| **23** | 67 | 5.6 | **60** | 23 | 4.8 |
| **24** | 63.3 | 5.6 | **61** | 21.6 | 6.6 |
| **25** | 61.8 | 4.6 | **62** | 22 | 6.7 |
| **26** | 62 | 5.4 | **63** | 21.4 | 7.2 |
| **27** | 61.7 | 5.9 | **64** | 20.2 | 4.3 |
| **28** | 62.6 | 6.8 | **65** | 19.3 | 6.7 |
| **29** | 62.8 | 6.9 | **66** | 19.3 | 6.8 |
| **30** | 62.8 | 7 | **67** | 18.7 | 7.2 |
| **31** | 62.3 | 7.2 | **68** | 17.9 | 4.5 |
| **32** | 60.8 | 4.2 | **69** | 16.7 | 3.4 |
| **33** | 59.9 | 6.5 | **70** | 15.7 | 4 |
| **34** | 61 | 7.1 | **71** | 16.2-16.3 | 4.4-5.1 |
| **35** | 60.2 | 6.5 | **72** | 16.6 | 6.5 |
| **36** | 60.3 | 7 | **73** | 15.8 | 7.1 |
| **37** | 59.9 | 4.4 | **74** | 15.5 | 7.5 |

| **Polypeptide spot no.** | **Molecular weight (M_r_ )** | **Isoelectric point (IP)** | **Polypeptide spot no.** | **Molecular weight (M_r_ )** | **Isoelectric point (IP)** |
| --- | --- | --- | --- | --- | --- |
| **75** | 17.2 | 8.2 | **84** | 5.1 | 4.2 |
| **76** | 17 | 9.6 | **85** | 10.5 | 4.9 |
| **77** | 11.9 | 5.4 | **86** | 9.2 | 6.2 |
| **78** | 12.7 | 6.9 | **87** | 9.6 | 9.8 |
| **79** | 12.3 | 7.1 | **88** | 10.3 | 4.6 |
| **80** | 12.2 | 7.3 | **89** | 9.2 | 5.6 |
| **81** | 11.1 | 4.5 | **90** | 3.8 | 3.1 |
| **82** | 10.8 | 6.6 | **91** | 4.1 | 9.7 |
| **83** | 10.4 | 3.1 |  |  |  |
