# Supplementary material for: Exploring Early and Late Toxoplasma gondii Strain RH Infection by Two-Dimensional Immunoblots of Chicken Immunoglobulin G and M Profiles
Source: PLoS One. 2015 Mar 24;10(3):e0121647. doi: 10.1371/journal.pone.0121647 (PMC4372353; doi:10.1371/journal.pone.0121647)
Supplement: S2 Table — Isoelectric point and molecular weight of shared immunogenic polypeptide spots using antibodies specific for IgG 56 dpi and 7dpi. (DOC) [file pone.0121647.s005.doc]

**Table S**2

| **Polypeptide spot no.** | **Molecular weight (M_r_ )** | **Isoelectric point (IP)** | **Polypeptide spot no.** | **Molecular weight (M_r_ )** | **Isoelectric point (IP)** |
| --- | --- | --- | --- | --- | --- |
| **1** | 351.5-355.8 | 6.3-6.6 | **38** | 61.3 | 4.5 |
| **2** | 327.7-330.9 | 6.6-6.8 | **39** | 61.3 | 4.6 |
| **3** | 309.2 | 5.4 | **40** | 61.4 | 6 |
| **4** | 110.8 | 4.1 | **41** | 62.6 | 6.8 |
| **5** | 106.3 | 4.3 | **42** | 62.8 | 6.9 |
| **6** | 102.7 | 4.5 | **43** | 62.8 | 7 |
| **7** | 99.2 | 5.3 | **44** | 62.3 | 7.2 |
| **8** | 100.7-101.5 | 5.8-6.4 | **45** | 61 | 6.8 |
| **9** | 103.8 | 6.6 | **46** | 61 | 7.1 |
| **10** | 93.7 | 7.2 | **47** | 60.9 | 7.3 |
| **11** | 96.8-98.3 | 6.6-6.7 | **48** | 60.8 | 7.5 |
| **12** | 88.7 | 6.9 | **49** | 60.6 | 9.7 |
| **13** | 82.8 | 7 | **50** | 60.8 | 4.2 |
| **14** | 100.8-102.4 | 4.3 | **51** | 59.9 | 4.4 |
| **15** | 96.9 | 4.4 | **52** | 55.8 | 4.5 |
| **16** | 92.4-92.7 | 4.5-5.7 | **53** | 60.2 | 6.5 |
| **17** | 90.2 | 6.2 | **54** | 60.3 | 6.9 |
| **18** | 85.1-87 | 3-4.3 | **55** | 60.3 | 7 |
| **19** | 75.7 | 3-4 | **56** | 59.7 | 7.5 |
| **20** | 78.2 | 4.3 | **57** | 59.8 | 9.8 |
| **21** | 73.4 | 4.3 | **58** | 47.2 | 4.2 |
| **22** | 74.1 | 4.4 | **59** | 37.5 | 4.4 |
| **23** | 74.8 | 4.7 | **60** | 47.6 | 5.7 |
| **24** | 74 | 5.7 | **61** | 40.5 | 6.5 |
| **25** | 76.8 | 6.5 | **62** | 50.6 | 7.1 |
| **26** | 75.5 | 7 | **63** | 51 | 7.2 |
| **27** | 78.9 | 9.8 | **64** | 36.3 | 5.7 |
| **28** | 69.7 | 4.2 | **65** | 37.6 | 6 |
| **29** | 66.5 | 4.3 | **66** | 29.2 | 5.5 |
| **30** | 69.6 | 4.5 | **67** | 29.4 | 5.8 |
| **31** | 68.5 | 5.8 | **68** | 30.1 | 6.5 |
| **32** | 68.1 | 7 | **69** | 30.1 | 7 |
| **33** | 69.7 | 7.1 | **70** | 29.7 | 7.2 |
| **34** | 67.4 | 7.1 | **71** | 29.5 | 7.4 |
| **35** | 67.1 | 7.2 | **72** | 30.8 | 7.5 |
| **36** | 60.9 | 3 | **73** | 30 | 8-8.2 |
| **37** | 66 | 4.2 | **74** | 26.7-28.3 | 9-10 |

| **Polypeptide spot no.** | **Molecular weight (M_r_ )** | **Isoelectric point (IP)** | **Polypeptide spot no.** | **Molecular weight (M_r_ )** | **Isoelectric point (IP)** |
| --- | --- | --- | --- | --- | --- |
| **75** | 29.5 | 3 | **103** | 15.8 | 7.1 |
| **76** | 27.2 | 4.1 | **104** | 15.5 | 7.5 |
| **77** | 28.3 | 4.4 | **105** | 12.7 | 6.9 |
| **78** | 26.4 | 4.5 | **106** | 12.3 | 7.1 |
| **79** | 27.2 | 4.5 | **107** | 12.2 | 7.3 |
| **80** | 27 | 4.8 | **108** | 10.8 | 6.6 |
| **81** | 24.6 | 4.3 | **109** | 10.8 | 6.9 |
| **82** | 23 | 4.8 | **110** | 9.6 | 9.8 |
| **83** | 21.6 | 6.6 | **111** | 10.3 | 3 |
| **84** | 22 | 6.7 | **112** | 10.4 | 3.1-3.3 |
| **85** | 21.4 | 7.2 | **108** | 10.8 | 6.6 |
| **86** | 24.1 | 7.5 | **109** | 10.8 | 6.9 |
| **87** | 23 | 3 | **113** | 10.4 | 4-4.4 |
| **88** | 22.3 | 4.3 | **114** | 9.2 | 5.6 |
| **89** | 20.2 | 4.3 | **115** | 9.2 | 6.2 |
| **90** | 17.9 | 4.5 | **116** | 9.4 | 4.1 |
| **91** | 19.3 | 6.7 | **117** | 7.1 | 3.1-3.6 |
| **92** | 19.3 | 6.8 | **118** | 7.4 | 4.4 |
| **93** | 18.9 | 7.1 | **119** | 8 | 6.5 |
| **94** | 18.7 | 7.2 | **120** | 6.8 | 6.5 |
| **95** | 18.9 | 7.3 | **121** | 7.3 | 6.6 |
| **96** | 18 | 7.5 | **122** | 6.1 | 8.3 |
| **97** | 17 | 9.6 | **123** | 5.2 | 9.8 |
| **98** | 20 | 3 | **124** | 4.4 | 4.4 |
| **99** | 16.7 | 3.4 | **125** | 5 | 4.6 |
| **100** | 15.7 | 4 | **126** | 3.8 | 6.5 |
| **101** | 16.2-16.3 | 4.4-5.1 | **127** | 3.6 | 8.8 |
| **102** | 16.6 | 6.5 | **128** | 4.1 | 9.7 |

| **Polypeptide spot no.** | **Molecular weight (M_r_ )** | **Isoelectric point (IP)** | **Polypeptide spot no.** | **Molecular weight (M_r_ )** | **Isoelectric point (IP)** |
| --- | --- | --- | --- | --- | --- |
| **150** | 4.5 | 6.9 | **153** | 4.4 | 4.2 |
| **151** | 4.3 | 6.6 | **154** | 4.1 | 4.4 |
| **152** | 4 | 7 | **155** | 4.2 | 6.8 |
