# Supplementary material for: Exploring Early and Late Toxoplasma gondii Strain RH Infection by Two-Dimensional Immunoblots of Chicken Immunoglobulin G and M Profiles
Source: PLoS One. 2015 Mar 24;10(3):e0121647. doi: 10.1371/journal.pone.0121647 (PMC4372353; doi:10.1371/journal.pone.0121647)
Supplement: S3 Table — Isoelectric point and molecular weight of shared polypeptide spots of 2-DE separated T. gondii strain RH tachyzoite proteins using antibodies specific for IgM 56 dpi and IgG 7 dpi. (DOC) [file pone.0121647.s006.doc]

**Table S3**

| **Polypeptide spot no.** | **Molecular weight (M_r_ )** | **Isoelectric point (IP)** | **Polypeptide spot no.** | **Molecular weight (M_r_ )** | **Isoelectric point (IP)** |
| --- | --- | --- | --- | --- | --- |
| **1** | 351.5-355.8 | 6.3-6.6 | **38** | 66.3 | 4.7 |
| **2** | 309.2 | 5.4 | **39** | 68.5 | 4.8 |
| **3** | 102.2 | 4.6 | **40** | 62 | 4.8 |
| **4** | 110.8 | 4.3 | **41** | 62 | 4.9 |
| **5** | 106.3 | 4.3 | **42** | 63.4 | 5.1 |
| **6** | 110.8 | 4.1 | **43** | 63.1 | 5.3 |
| **7** | 106.3 | 4.3 | **44** | 63.1 | 5.5 |
| **8** | 105.7 | 4.8 | **45** | 62.5 | 7.1 |
| **9** | 100.8-102.4 | 4.3 | **46** | 62.3 | 7.2 |
| **10** | 102.7 | 4.5 | **47** | 61 | 4.5 |
| **11** | 96.9 | 4.4 | **48** | 61.9 | 5 |
| **12** | 99.2 | 5.3 | **49** | 61.8 | 5.3 |
| **13** | 100.7-101.5 | 5.8-6.4 | **50** | 61 | 7.1 |
| **14** | 96.8-98.3 | 6.6-6.7 | **51** | 61 | 4.2 |
| **15** | 92.4-92.7 | 4.5-5.7 | **52** | 60.9 | 5 |
| **16** | 85.5-86 | 4.2-4.4 | **53** | 60.7 | 5.5 |
| **17** | 87.2 | 4.9 | **54** | 60.65 | 5.8 |
| **18** | 90.2 | 6.2 | **55** | 60.6 | 6.6 |
| **19** | 88.7 | 6.9 | **56** | 60.6 | 6.9 |
| **20** | 82.2 | 4.4 | **57** | 60.6 | 7.1 |
| **21** | 85.5 | 5.4 | **58** | 47.2 | 4.2 |
| **22** | 79.7-80.6 | 5-5.1 | **59** | 59.9 | 4.4 |
| **23** | 82.8 | 7 | **60** | 55.8 | 4.5 |
| **24** | 78.1 | 4.2-4.3 | **61** | 59.5 | 4.9 |
| **25** | 74.7 | 4.2 | **62** | 60.4 | 5.1 |
| **26** | 74.7 | 4.3 | **63** | 50.6 | 7.1 |
| **27** | 74.3 | 4.5 | **64** | 51 | 7.2 |
| **28** | 73.4 | 4.6 | **65** | 36.3 | 4.1 |
| **29** | 73.5 | 4.7 | **66** | 37.5 | 4.4 |
| **30** | 73.5 | 5.1 | **67** | 36.3 | 5 |
| **31** | 75.8 | 5.8 | **68** | 47.6 | 5.7 |
| **32** | 69.7 | 4.5 | **69** | 36.3 | 5.7 |
| **33** | 69.6 | 4.7 | **70** | 52.1 | 5.3 |
| **34** | 73.4 | 5.3 | **71** | 37.6 | 6 |
| **35** | 69.7 | 6 | **72** | 40.5 | 6.5 |
| **36** | 68.5 | 5.2 | **73** | 46.7 | 5.5 |
| **37** | 65.3 | 4.4 | **74** | 35 | 4.1 |

| **Polypeptide spot no.** | **Molecular weight (M_r_ )** | **Isoelectric point (IP)** | **Polypeptide spot no.** | **Molecular weight (M_r_ )** | **Isoelectric point (IP)** |
| --- | --- | --- | --- | --- | --- |
| **75** | 33.4 | 4.1 | **111** | 15.8 | 7.1 |
| **76** | 28.3 | 4.4 | **112** | 15.5 | 7.5 |
| **77** | 33.4 | 4.5 | **113** | 16.8 | 8.5 |
| **78** | 35 | 4.7 | **114** | 26.7-28.3 | 9-10 |
| **79** | 33.7 | 4.8 | **115** | 9.8 | 3.1-3.3 |
| **80** | 29.2 | 5.5 | **116** | 9.8 | 4-4.4 |
| **81** | 39.1 | 5.2 | **117** | 12 | 4.1 |
| **82** | 41.2 | 5.5 | **118** | 9.4 | 4.7 |
| **83** | 41.1 | 5.9 | **119** | 10.7 | 5 |
| **84** | 41.2 | 7.2 | **120** | 11.8 | 5.3 |
| **85** | 45.7 | 7.7 | **121** | 11.4 | 4.8 |
| **86** | 30 | 8-8.2 | **122** | 10.6 | 5 |
| **87** | 26.7-28.3 | 9-10 | **123** | 11.4 | 5.4 |
| **88** | 29.2 | 4.4 | **124** | 12 | 6.1 |
| **89** | 29 | 4.5 | **125** | 9.9 | 6.3 |
| **90** | 29.2 | 4.6 | **126** | 10.8 | 6.6 |
| **91** | 27 | 4.8 | **127** | 12.7 | 6.9 |
| **92** | 33.3 | 6.6 | **128** | 12.3 | 7.1 |
| **93** | 32.5 | 6.6 | **129** | 12.2 | 7.3 |
| **94** | 19.3 | 4.1 | **130** | 12.2 | 9.8 |
| **95** | 20.2 | 4.3 | **131** | 11.1 | 9.5 |
| **96** | 22.8 | 5.4 | **132** | 9.2 | 5.3 |
| **97** | 23.4 | 5.7 | **133** | 9.4 | 5 |
| **98** | 21.6 | 6.6 | **134** | 8.6 | 5.9 |
| **99** | 22 | 6.7 | **135** | 9.2 | 6.2 |
| **100** | 21.4 | 7.2 | **136** | 9.8 | 7.1-7.5 |
| **101** | 17.9 | 4.5 | **137** | 9.8 | 8.94 |
| **102** | 19.3 | 6.7 | **138** | 9.6 | 9.8 |
| **103** | 19.3 | 6.8 | **139** | 8.5 | 10 |
| **104** | 18.7 | 7.2 | **140** | 6.58 | 3 |
| **105** | 17 | 9.6 | **141** | 6.5 | 3.1-3.6 |
| **106** | 16.1 | 3.5 | **142** | 6.05 | 4-4.5 |
| **107** | 15.7 | 4 | **143** | 5 | 4.6 |
| **108** | 15.9 | 4.4 | **144** | 6.8 | 6.5 |
| **109** | 16.9 | 6 | **145** | 3.8 | 6.5 |
| **110** | 16.2 | 6.3 |  |  |  |
